# Clinicopathological Correlation and Recurrence Outcome of Adnexal Involvement on Primary Extramammary Paget’s Disease (EMPD)

**DOI:** 10.7759/cureus.61975

**Published:** 2024-06-08

**Authors:** Sabita Aryal, Subodh Bashyal, Liu Ye Qiang

**Affiliations:** 1 Department of Dermatology and Venereology, Shanghai Skin Disease Hospital, Tongji University School of Medicine, Shanghai, CHN; 2 Department of Endocrinology and Metabolism, Shanghai Tenth People's Hospital, Tongji University School of Medicine, Shanghai, CHN; 3 Department of Dermatology, Shanghai Skin Disease Hospital, Tongji University, Shanghai, CHN

**Keywords:** clinicopathological correlation, diagnosis, prognosis, paget’s disease, primary empd, neoplasms, adnexal and skin appendage

## Abstract

Introduction

Primary extramammary Paget's disease (EMPD) is a rare neoplasm that manifests as well-defined erythematous plaques, often misdiagnosed due to its similarity with different dermatoses. It may exhibit invasive features, involving adnexal invasions. The study aims to assess and compare the clinicopathological correlation of primary EMPD with adnexal features.

Materials and methodology

The monocentric observational retrospective study observed 43 confirmed primary EMPD cases in patients aged 45-95, excluding those with infectious dermatoses, pseudo-tumors, secondary lesions, or survived less than a month. Demographical, clinical and pathological observations were recorded. Expert dermatopathologists, blinded to the initial diagnosis, conducted a comprehensive histopathological evaluation yielding differential pathological diagnosis.

Statistical analysis involved Pearson’s Chi-square, Mann-Whitney U, and Spearman’s Correlations for clinicopathological concordance and adnexal features. Recurrence was evaluated using Kaplan-Meier and log-rank tests, while multivariate recurrence analyses include Cox regression. A p-value < 0.05 was deemed significant.

Results

There was a significant association between adnexal involvement and the site of lesion (p < 0.05). There was a significant association (p < 0.05) between involved adnexal depth and primary EMPD subtypes. Adnexal involvement has a significant association with the concordance rates derived from clinicopathological correlations (p < 0.05). Smaller lesions and non-invasive EMPD significantly predict longer recurrence onset (p < 0.01). The primary EMPD subtype was the only independent predictor for recurrence time using the Cox regression model.

Conclusion

Adnexal proliferation in primary EMPD is considered vital on clinicopathological correlations and recurrence predictions, suggestive of its utility on both diagnosis and prognosis.

## Introduction

In 1874, James Paget first identified a condition presenting as eczema-like lesions around the breast and areola, which later progressed into breast cancer. Following this, extramammary Paget’s disease (EMPD) emerged in 1889 when Crocker documented similar lesions in the penile and scrotal areas, exhibiting histological and clinical patterns similar to mammary Paget’s disease (MPD). Primary EMPD, characterized by distinct erythematous plaques that can evolve into pruritic, eczematous lesions with characteristic white "cake-icing" scaling or ulcerations, represents a rare cutaneous neoplasm primarily occurring in apocrine gland-rich regions [1,2]. The resemblance of its clinical presentation to more common dermatoses often leads to misdiagnosis, so it requires histopathological and immunohistochemical analyses for early, accurate diagnosis and differentiation from secondary EMPD and other similar cancers. Utilizing clinicopathological correlations is pivotal in refining diagnostic precision and tailoring therapeutic interventions. Treatment typically involves surgical excision, with non-surgical options available for certain cases [2,3]. Furthermore, assessing adnexal involvement and the depth of invasion has been recognized as crucial for prognostication in EMPD [4,5], underscoring the importance of these parameters in the comprehensive understanding and management of this elusive disease. By comparing the clinical features and consensus derived from clinical and pathological diagnoses and the features of adnexal involvement, this study seeks to understand the diagnostic and prognostic significance of adnexal features in primary EMPD.

## Materials and methods

Data sources and study population

This monocentric observational retrospective study spanning from January 1, 2022 to December 31, 2023 encompassed 43 confirmed cases of primary EMPD across both genders - male and female, with patient ages ranging from 45 to 95 at Shanghai Skin Disease Hospital, Shanghai. The location of the study was chosen due to its ease of access to the inpatient and outpatient department, excellent medical experts of the topic under study, the availability of laboratory facilities, and the hospital being a special center for skin conditions, especially, skin neoplasms in Shanghai.

Exclusion criteria for the study included individuals presenting with infectious dermatoses, pseudo-tumors, secondary lesions, or patients with a survival time of less than one month. Patient participation was secured only through signed informed consent, in accordance with the approval from the hospital's ethical committee.

Demographic, clinical, pathological and three-year recurrence onset data were obtained from medical and follow-up records. Pathological observations were documented. Local recurrence was classified as the emergence of a tumor at the initial site following a disease-free interval of at least six months.

Concordance derived from clinicopathological correlation

Expert dermatopathologists, blinded to the initial diagnosis to mitigate bias, conducted a comprehensive histopathological evaluation providing a subjective differential pathological diagnosis. Concordance was derived from clinicopathological correlations based on the agreement between clinical differential diagnosis and pathological differential diagnosis, labeled as "concordant" when they agreed and "discordant" when they did not.

Statistical analysis

Statistical analysis in this study involves employing Chi-square tests, or Fisher’s exact test where appropriate, to examine the relationships between demographical and clinicopathological observations, differential diagnoses, primary EMPD subtypes in relation to adnexal involvement. Additionally, the Mann-Whitney U test is utilized to compare the depth of adnexal involvement across different primary EMPD subtypes, evaluating its utility as a prognostic marker.

To evaluate the diagnostic accuracy and the alignment between clinical assessments and pathological results, concordance rates from clinicopathological correlations were calculated. Reliability analysis was undertaken, utilizing Cohen's kappa statistics, to quantitatively measure the agreement beyond chance between clinical and pathological differential diagnoses.

Spearman correlation was used to find the association between adnexal features of depth and involvement with the concordance derived from clinicopathological correlations. Further, to assess the association of adnexal involvement between clinical and pathological differential diagnoses, z-test of difference in concordance ratios was calculated across groups differentiated by adnexal involvement status.

Recurrence rates across a three-year period were assessed using Kaplan-Meier survival curves and further evaluated through the log-rank test for significance. To identify factors independently associated with recurrence, both univariate and multivariate analyses were conducted employing the Cox proportional hazards regression model.

Statistical analyses were performed with a significance threshold set at (p < 0.05), and outcomes are presented alongside 95% confidence intervals (CIs) where relevant.

## Results

**Figure 1:**
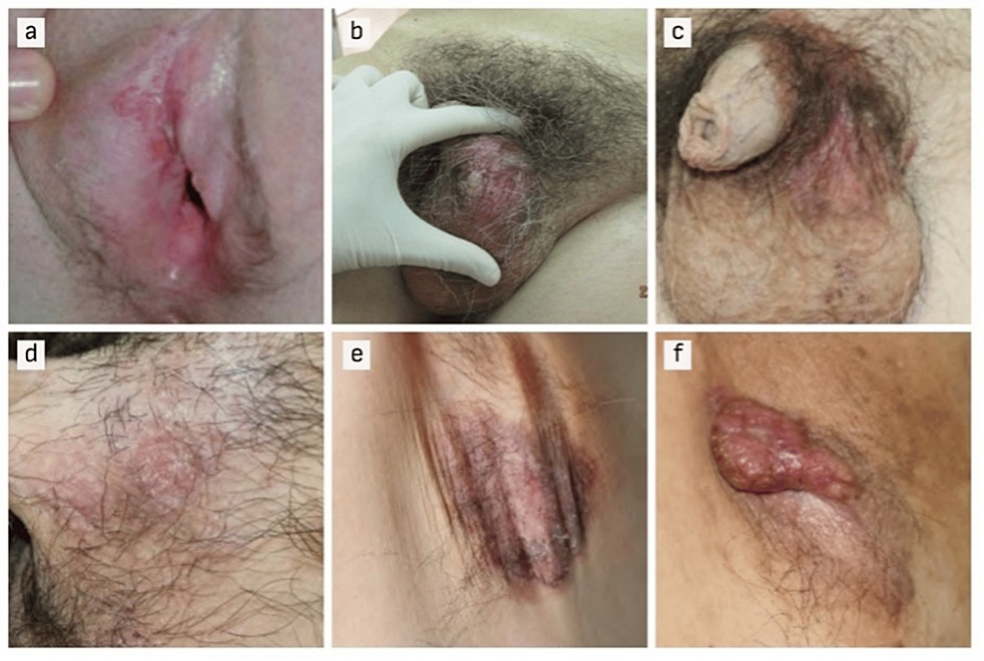
Skin lesions show clinical manifestations of primary EMPD at (a) vulvar region, (b, c) scrotal region, (d) penoscrotal region, (e, f) axillary region.

The mean age of 43 patients under study was 68.91 years (SD = 10.8), with a predominant male representation (88.4%) compared to females (11.6%). The gender-stratified analysis revealed no significant difference in mean age between males and females (t = -0.717, df = 41, p = 0.448). The distribution of primary EMPD across various sites (Figure 1) revealed a predominant occurrence in the penoscrotal region (69.8%), followed by the vulvar region (11.6%). Approximately one-fifth (20.0%) of the cases were noted in the perineal, axillary, and genital regions. Clinical features were diverse, predominantly, 74.4% of cases exhibited erythema. Erosion or ulceration and pruritus were observed in 60.5% of cases each, while hypopigmentation was present in 18.6% of cases. Eczematous presentations were noted in 14.0% of cases. Clinical diagnosis made during initial patient consultations obtained from the medical records revealed primary EMPD being the most diagnosed at 55.8% followed by eczema in 16.3% of cases, trunk skin infection in 14.0%, unknown diagnoses in 9.3%, and Paget-like Bowen’s disease in 4.7%.

**Figure 2:**
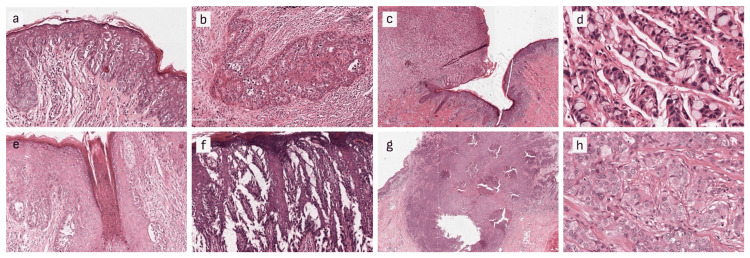
Pathological manifestations of primary EMPD (100X HE Stains) of Paget cells show (a) limited to the epidermis; (b) invading dermis; (c) invading hair follicles; (d) causing acantholysis; Paget cells are diffusely distributed throughout the epidermis and middle and upper dermis in a gland-like arrangement (e): HE, ×10, (f): HE, ×200; Paget cells are distributed in nodules in the dermis (g) HE, ×10, (h) HE, ×200

Several noteworthy pathological findings were observed along with the pagetoid cells in the lesions (Figure 2). The majority of cases presented adnexal involvement (83.7%). Other notable pathological features were keratinization of the tissues with parakeratosis (72.1%), and hyperkeratosis (69.8%). Cellular atypia was noted in 48.8% of cases, while acantholysis was observed in 23.3%.

**Figure 3:**
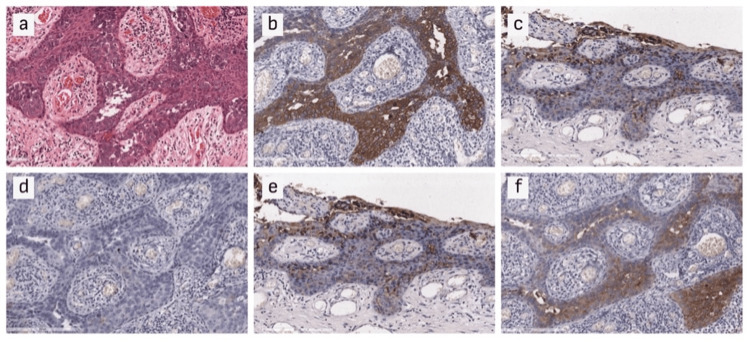
Immunohistochemical staining of tissues (a: HE staining, ×100; b: CK7 staining, ×100; c: CK20 staining, ×100; d: CEA staining, ×100; e: EMA staining, ×100; f: GCDFP-15 staining, ×100)

Immunohistochemical testing revealed distinctive patterns, as shown in Figure 3, with the majority of cases exhibiting positive staining for CK7 (94.0%) out of 36 tests, EMA (89.0%) out of 36 tests, CEA (71.0%) out of 34 and PAS (89.0%) out of 18 tests. Conversely, negative staining was observed for CK20 (90.0%) out of 40 tests, and GCDPF-15 (50.0%) out of 26 tests. The positive test results of CK7 and GCDFP-15 coupled with the negative test results of CK20 were instrumental in differentiating primary and secondary EMPD, although with very low sufficiency. Similarly, other supplementary tests like CEA, PAS, Melan A, HMB45, etc. were carried out to rule out mimicking carcinoma or dermatoses. A comprehensive analysis revealed that the non-invasive subtype constituted the majority subtype with 33 cases (76.7%), while the invasive subtype accounted for 10 cases (23.3%).

The blinded thorough pathological analysis, conducted without access to clinical details, revealed primary EMPD as the predominant diagnosis accounting for 81.4% of cases (35 cases) followed by Paget-like Bowen's disease in 11.6% (five cases) and malignant melanoma in 7.0% (three cases).

Description of adnexal features

The analysis of adnexal features included prevalence and depth of adnexal involvement among various types of adnexal structures of sebaceous glands, hair follicles, and different sweat gland components - apocrine glands and ducts, eccrine glands and ducts, and unknown ducts, as shown in Table 1.

**Table 1:** Involvement and presence of different adnexal structures along with the depth of infiltration

Types of Adnexa	Involved N (%)	Present N (%)	Depth of Adnexal Involvement (mm)
Sebaceous Glands	13 (38%)	34 (79%)	0.4–1.3 (mean 0.92)
Hair Follicles	36 (86%)	42 (98%)	0.6–2.8 (mean 1.54)
Sweat Glands			
Apocrine Glands & Ducts	1 (4%)	26 (60%)	1.6 (mean 1.6)
Eccrine Glands & Ducts	36 (86%)	37 (86%)	0.8–3.1 (mean 2.5)
Unknown Ducts	1 (3%)	30 (70%)	2.4 (mean 2.4)

Sebaceous glands, present in 79% of the cases, showed involvement in 38% of them, with the depth of involvement ranging around 0.92 mm on average. Hair follicles, found in nearly all samples, had a higher involvement rate of 86%, and a deeper average penetration of 1.54 mm. Apocrine glands and ducts, while present in 60% of the cases, were only involved in 4% of them, with a mean depth of 1.6 mm. Similarly, eccrine glands and ducts, found in 86% of the cases, also had a high involvement rate of 86% but with the deepest average invasion of 2.5 mm. Additionally, an unknown type of duct present in 70% of the cases was involved in 3% of them, with an involvement depth of 2.4 mm.

Clinicopathological correlation of adnexal involvement status

Chi-square tests of association (Fisher’s Exact Test, if applicable) employed between clinicopathological features, subtypes of primary EMPD with the involvement status of adnexa, resulted in findings (Table 2), and are summarized as follows:

Demographics

The mean age did not significantly differ between cases with adnexal involvement (M = 70.29, SD = 13.51) and those without (M = 68.4, SD = 9.62; p = 0.700). There was no significant association between gender and adnexal involvement (p > 0. 05).

Clinical Findings

Clinical observations including areas with apocrine sweat glands, presence of inflammation and swelling, eczematous features, pruritus, erythema, lesion area and differential clinical diagnoses did not show significant associations with adnexal involvement (p > 0.05). There was a significant association between the involvement of adnexal structures and the presence of lesions in the axillary, penoscrotal, genital, perineal, and vulvar regions (p < 0.05).

Pathological and Immunohistochemical Findings

Pathological features of parakeratosis, hyperkeratosis and cellular atypia, acantholysis did not exhibit significant associations with adnexal involvement (p > 0.05). None of the immunohistochemical tests was found to be significantly associated with adnexal involvement (p > 0.05).

Diagnostic Findings

There were no significant associations between adnexal involvement and either clinical or pathological differential diagnoses. Moreover, despite the commonality of adnexal involvement, it showed no significant association with the invasion level of primary EMPD, indicated by p-values greater than 0.05.

**Table 2:** Comparison of adnexal involvement with demographic, clinical, pathological, immunohistochemical, diagnostic observations and findings ^f^p-value: Fisher’s exact test ^t^p-value: t-test

Parameters			Adnexal Involvement		p-value
			Yes (n=36)	No (n=7)	
General Demographics	Age (years) Mean ± SD		70.29 ± 13.51	68.4 ± 9.62	0.700 ^t^
	Gender	Male	6 (85.7%)	32 (88.9%)	0.608 ^f^
		Female	1 (14.3%)	4 (11.1%)	
Clinical Findings	Erythema	Yes / Present	28 (77.8%)	4 (57.1%)	0.347 ^f^
		No / Absent	8 (22.2%)	3 (42.9%)	
	Eczematous	Yes / Present	5 (13.9%)	1 (14.3%)	1.000 ^f^
		No / Absent	31 (86.1%)	6 (85.7%)	
	Erosions or ulcerations	Yes / Present	21 (58.3%)	5 (71.4%)	0.685 ^f^
		No / Absent	15 (41.7%)	2 (28.6%)	
	Pruritus	Yes / Present	21 (58.3%)	5 (71.4%)	0.685 ^f^
		No / Absent	15 (41.7%)	2 (28.6%)	
	Hypopigmentation	Yes / Present	7 (19.4%)	1 (14.3%)	1.000^ f^
		No / Absent	29 (80.6%)	6 (85.7%)	
	Lesion Area (cm^2^) ± Median (IQR)		8.5 (14.75)	10 (17)	0.733 ^t^
	Differential clinical diagnosis	Eczema	4 (11.1%)	3 (42.9%)	0.100^ f^
		Trunk Skin Infection	4 (11.1%)	2 (28.6%)	
		Unknown Diagnosis	4 (11.1%)	0 (0%)	
		EMPD	22 (61.1%)	2 (28.6%)	
		Bowen’s Disease	2 (5.6%)	0 (0%)	
	Site of the lesion	Penoscrotal	27 (75.0%)	3 (42.9%)	0.019 ^f^
		Axillary	1 (2.8%)	2 (28.6%)	
		Vulvar	0 (0%)	5 (13.9%)	
		Genital	0 (0%)	1 (14.3%)	
		Perineal	3 (8.3%)	1 (14.3%)	
Pathological Findings	Hyperkeratosis	Yes / Present	25 (69.4%)	5 (71.4%)	1.000 ^f^
		No / Absent	11 (30.6%)	2 (28.6%)	
	Parakeratosis	Yes / Present	26 (72.2%)	5 (71.4%)	1.000 ^f^
		No / Absent	10 (27.8%)	2 (28.6%)	
	Cellular Atypia	Yes / Present	17 (47.2%)	4 (57.1%)	0.698 ^f^
		No / Absent	19 (52.8%)	3 (42.9%)	
	Acantholysis	Yes / Present	9 (25%)	1 (14.3%)	1.000 ^f^
		No / Absent	27 (75%)	6 (85.7%	
	Differential Pathological Diagnosis	Bowen’s Disease	2 (5.6%)	0 (0.0%)	0.489 ^f^
		Primary EMPD	30 (83.3%)	5 (71.4%)	
		Malignant Melanoma	4 (11.1%)	2 (28.6%)	
Immunohistochemical Findings	CK20-		31 (86.1%)	5 (71.4%)	0.318 ^f^
	CK7+		30 (83.3%)	4 (57.1%)	0.470 ^f^
	EMA+		27 (75%)	5 (71.4%)	0.586 ^f^
	S100-		16 (44.4%)	3 (42.9%)	0.635 ^f^
	GCDPF15-		12 (33.3%)	1 (14.3%)	0.303 ^f^
	HMB 45-		8 (22.2%)	2 (28.6%)	0.524 ^f^
	CEA+		20 (55.6%)	4 (57.1%)	0.635 ^f^
	Melan A-		8 (22.2%)	2 (28.6%)	0.524 ^f^
Diagnostic Findings	Subtype of EMPD	Type 1a (In Situ)	27 (75%)	6 (85.7%)	0.302 ^f^
		Type 1b (Invasive)	9 (25%)	1 (14.3%%)	

Prognostic correlation of adnexal depth

The Mann-Whitney test (Table 3) revealed a significant difference in adnexal depth between non-invasive (Mean Rank: 17.36) and invasive (Mean Rank: 21.08) groups (p < 0.001). This finding suggests that invasive EMPD is associated with greater adnexal depth, indicating a higher likelihood of invasiveness with increased adnexal involvement.

**Table 3:** Correlation between adnexal depth & EMPD subtypes ^⧺^p-value: p-values calculated using Mann-Whitney U test (Exact 2-sided Sig.)

	EMPD Sub Types	Count	Mean Rank	Sum of Ranks	p-value
Adnexal Depth (mm)	Non-Invasive	27	15.35	414.50	.001 ^#^
	Invasive	9	27.94	251.50	
	Total	36			

Analysis of concordance derived from clinicopathological correlations

The pathological blind evaluation of the clinically diagnosed cases, as shown in Table 4, was as follows: Among 29 cases clinically suspected of primary EMPD, pathological differential diagnosis revealed 22 to be EMPD, three as Bowen's disease, and four as malignant melanoma. Pathological diagnosis of two cases initially suspected of Bowen’s disease was considered to be EMPD. Similarly, among four cases of eczema diagnosed clinically, three cases were considered EMPD with a single case of Bowen’s disease. Similarly, five cases clinically suspected of trunk skin infection were considered EMPD in four and malignant melanoma in one case. Lastly, among five cases of unknown diagnosis, four cases were considered EMPD, and one case was considered Bowen’s disease.

**Table 4:** Confusion matrix of histopathological diagnosis and clinical diagnosis

Adnexal Involvement	Clinical Diagnosis	Histopathological Diagnosis			Row Total	Concordance Rates
		BD	EMPD	MM		
Cases without adnexal involvement (n=7)	Eczema	1	1		2	14.3%
	EMPD	1	1	2	4	
	TSI		1		1	
	Column Total	2	4	1	7	
Cases with adnexal involvement (n=36)	Bowen's Disease		2		2	58.3%
	Eczema		2		2	
	EMPD	2	21	2	25	
	TSI		3	1	4	
	Unknown	1	2		3	
	Column Total	3	30	3	36	
All Cases (n=43)	Bowen's Disease		2		2	51.2%
	Eczema	1	3		4	
	Primary EMPD	3	22	4	29	
	TSI		4	1	5	
	Unknown	1	4		3	
	Column Total	5	33	3	43	

A concordance rate of 51.2% was observed across all cases, with a notably lower concordance of 14.3% in cases without adnexal involvement and a higher rate of 58.3% in cases with adnexal involvement. Statistical analysis using Fisher’s Exact test and a Mann-Whitney U-test indicated a significant difference in concordance rates related to adnexal involvement (p < 0.05), underscoring the importance of considering adnexal features in the accurate diagnosis of EMPD (Table 5).

**Table 5:** Cross-tabulation of diagnostic concordance with and without adnexal involvement

	Adnexal Involvement		
Clinicopathological Concordance	Yes	No	p-value
Concordant	21	1	.046
Discordant	15	6	

Recurrence analysis of primary EMPD

**Figure 4:**
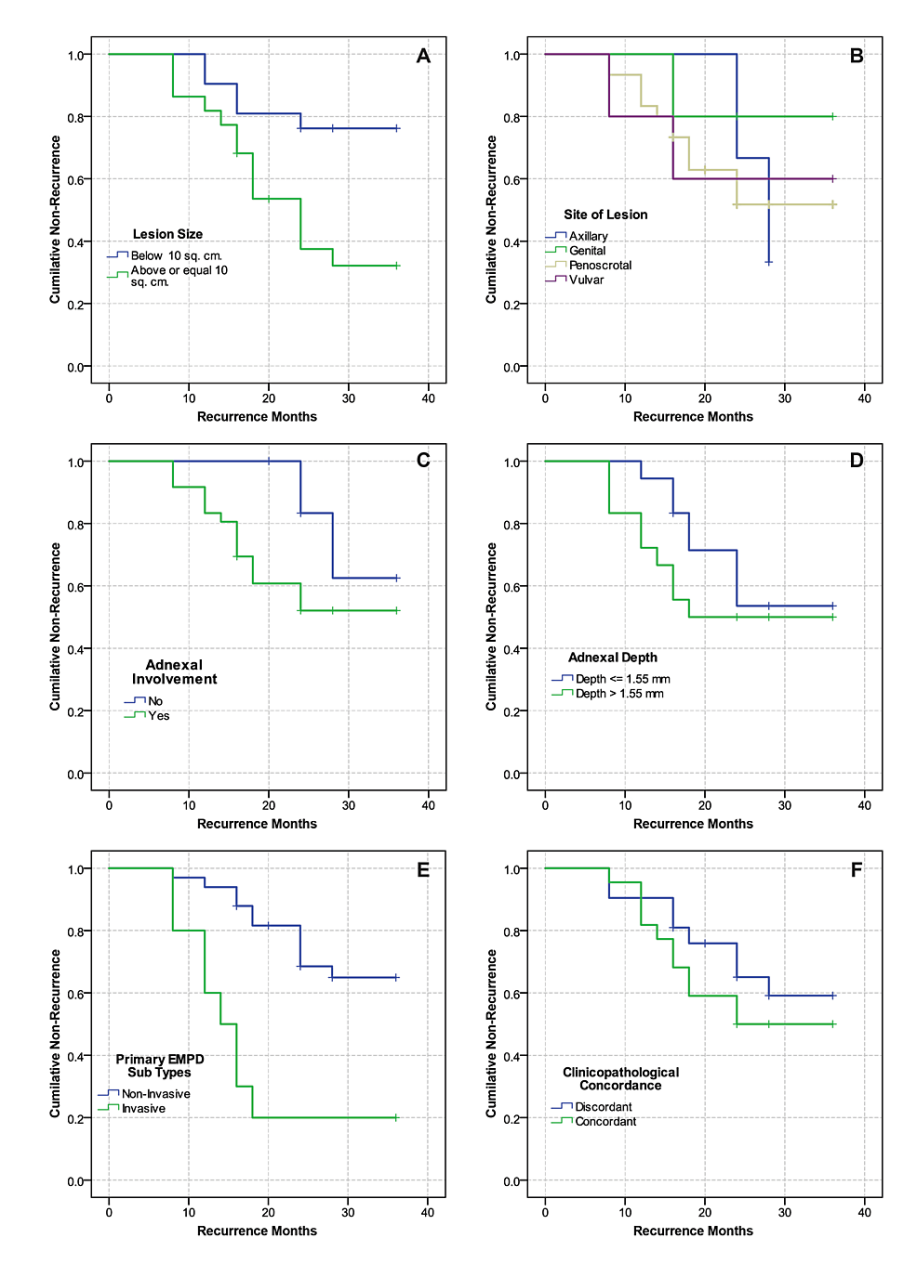
The Kaplan-Meier survival plots demonstrate the three-year local recurrence in (A) size of the lesion, (B) site of the lesion, (C) adnexal involvement, (D) adnexal depth, (E) EMPD subtype and (F) clinicopathological concordance

Recurrence rates over three years were evaluated using Kaplan-Meier survival analysis (Table 6), the log-rank test, and Cox regression models, considering various clinicopathological factors. The analysis, illustrated in Figure 4, revealed that patients with lesion sizes of 10 cm² or smaller had a longer mean recurrence-free survival time of 31.2 months, compared to 23.3 months for patients with lesions larger than 10 cm², indicating a significant difference (χ² = 6.981, df = 1, p = 0.008). Moreover, patients with invasive EMPD experienced a significantly shorter recurrence-free interval of 17.6 months versus those with non-invasive EMPD (χ² = 13.313, df = 1, p < 0.001). However, factors such as lesion site, adnexal involvement, adnexal depth, and clinicopathological concordance did not significantly affect recurrence times (p > 0.05).

**Table 6:** Kaplan-Meier analysis of univariates for the association of recurrence of primary EMPD

Parameters of Kaplan-Meier Analysis for Recurrence	Estimate	Std. Error	95% Confidence Interval		Chi-Square	Sig.
			Lower Bound	Upper Bound		
Site of Lesion						
Axillary	26.667	1.540	23.649	29.684	1.213	0.750
Genital	32.000	3.578	24.988	39.012		
Penoscrotal	26.450	1.948	22.631	30.269		
Vulvar	26.400	5.378	15.858	36.942		
Overall	27.291	1.589	24.177	30.405		
Size of Lesion						
Below 10 sq. cm	31.238	1.917	27.482	34.994	6.981	.008
Above or equal to 10 sq. cm	23.286	2.217	18.941	27.631		
Overall	27.291	1.589	24.177	30.405		
Adnexal Involvement						
No	32.333	2.097	28.223	36.444	1.085	.298
Yes	26.229	1.808	22.686	29.773		
Overall	27.291	1.589	24.177	30.405		
Adnexal Depth						
Depth <= 1.55 mm	28.159	2.138	23.969	32.349	.595	.441
Depth > 1.55 mm	24.222	2.841	18.654	29.791		
Overall	26.229	1.808	22.686	29.773		
Clinicopathological Concordance						
Discordant	28.744	2.172	24.486	33.001	.625	.429
Concordant	25.909	2.269	21.462	30.356		
Overall	27.291	1.589	24.177	30.405		
Primary EMPD Subtype						
Non-Invasive	30.227	1.514	27.260	33.194	13.313	.000
Invasive	17.600	3.070	11.583	23.617		
Overall	27.291	1.589	24.177	30.405		

**Figure 5:**
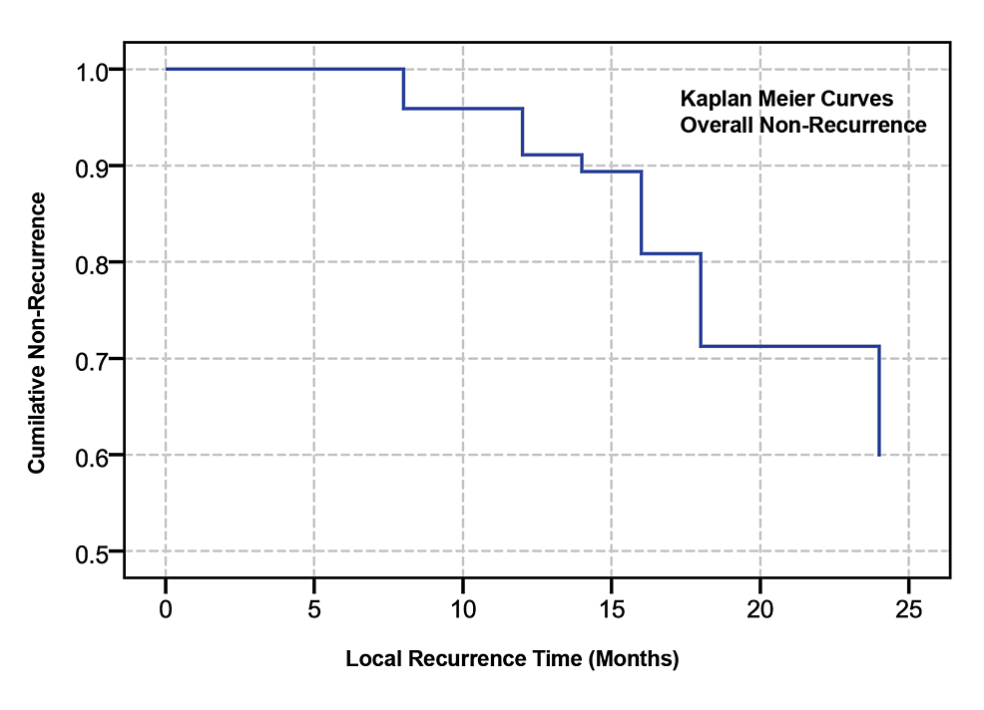
Overall local recurrence as predicted by Cox model

Multivariate analysis using Cox proportional hazards regression analysis (Table 7) revealed primary EMPD invasion level as the only independent predictor of recurrence time (χ² = 15.811, df = 7, p = 0.027). Patients with invasive EMPD had a significantly higher risk of recurrence compared to those with non-invasive EMPD. The hazard ratio (HR) of 7.636 indicated that the risk of recurrence for patients with invasive EMPD was approximately 7.6 times that of patients with non-invasive EMPD. This finding was statistically significant (Wald statistic = 6.692, p = 0.010). None of the other variables examined were predictive of recurrence within three years (p > 0.05). The local recurrence after 24 months was predicted to be 40% (Figure 5).

**Table 7:** COX regression model for multivariate analysis of recurrence

Covariates for COX Regression	B	SE	Wald	df	Sig.	Exp(B)	95.0% CI for Exp(B)	
							Lower	Upper
Site of Lesion			.330	3	.954			
Penoscrotal	-12.571	762.689	.000	1	.987	.000	.000	.
Vulvar	-.713	1.273	.314	1	.576	.490	.040	5.942
Perianal	-.123	.829	.022	1	.882	.885	.174	4.491
Lesion Area (cm^2^)	.035	.026	1.874	1	.171	1.036	.985	1.090
Depth of Involved Adnexa (mm)	-.309	.355	.758	1	.384	.734	.366	1.473
Clinicopathological Concordance	.473	.654	.523	1	.470	1.604	.446	5.778
Sub Types	2.033	.786	6.692	1	.010	7.636	1.637	35.629

## Discussion

In this study, no significant age difference between genders was observed, aligning with previous small-scale studies on EMPD, which similarly reported higher occurrence in the elderly, with a slight male predominance [5]. In a study by Kang et al. [6], the median age at first diagnosis was 71.0 years, and the peak incidence was between 60 and 80 years (65.1%). This aligns with this study's average age of 68.91 ± 10.18 years. However, the predominant male representation (88.4%) compared to females (11.6%) in this study is contrary to findings of other similar studies [3,7,8], suggesting potential variations in age demographics among EMPD cases owing to the fact that the cases in this study were very low (n = 43). Clinical presentations displayed diverse patterns with lesion sites revealing high occurrences in the penoscrotal region. In a similar small-scale study by Son et al. [9], primary EMPD was mostly present in the penoscrotal region in older males. However, few patients presenting with eczematous symptoms reported persistent eczema-like presentations over the long term (>16 months). Clinicians must conduct critical investigations of long-term eczematic presentations at the lesion sites discussed above, along with skin biopsies, for accurate diagnosis [3,10].

The clinical misdiagnosis rate (44.2%) was notably high, possibly attributed to factors such as asymptomatic presentations or mild symptoms, making it challenging to require critical attention. This often complicates case diagnosis and introduces the possibility of errors, especially when dealing with similar dermatoses [11]. Additionally, the lesions of this disease exhibited similarities with inflammatory dermatoses like eczema and tinea cruris, leading to potential confusion. Also, given the rarity of this disease, clinicians, perhaps due to limited experience, may need to be more aware of the case under scrutiny. Pathological analysis demonstrated the most prevalent features as adnexal involvement (83.7%) followed by keratinization of the tissues with parakeratosis (72.1%) and hyperkeratosis (69.8%), respectively. Cellular atypia (48.8%) and acantholysis (23.3%) were also present. A pathological examination of adnexal infiltration in various skin structures revealed a mean infiltration depth of 2.25 mm (SD = 0.46), ranging from 1.5 mm to 3.1 mm. Hair follicles and eccrine glands were the most frequently and deeply affected. In some cases, neoplastic cells replaced myoepithelial cells in adnexal structures, yet no underlying carcinoma was found. This aligns with studies advocating a meticulous examination of adnexal structures in EMPD cases [12].

Immunohistochemical markers are valuable constructs in the potential diagnosis of EMPD. Positive staining for CK7 (94.4%), EMA (88.8%), and CEA (70.5%) was prevalent, aligning with existing literature on EMPD marker expression [13]. However, the absence of significant associations between certain markers and adnexal involvement emphasizes the complexity of EMPD's immunohistochemical profile in relation to adnexal features. Immunohistochemistry can be used to distinguish primary and secondary EMPD, as well as other skin tumors with Paget-like morphological spread, such as Paget-like Bowen's disease, amelanotic melanoma, Paget-like reticulosis, etc. [14]. CK7 is a sensitive biomarker of EMPD, and CK20 is often expressed in gastrointestinal and urinary tract tumor cells. Therefore, primary EMPD is often CK7 positive and CK20 negative, while secondary EMPD is both positive [15]. In this study, the positive rate of CK7 was 94.4% (34/36 cases), and the positive rate of CK20 was also 19.4% (7/36 cases). Therefore, there are still shortcomings in distinguishing primary and secondary EMPD with CK20. GCDFP-15 is a marker protein of the apocrine sweat gland, often expressed positively in EMPD [16]. In this study, the positivity rate of GCDFP-15 was 51.9%, although insufficient to differentiate cases on subtypes. Previous studies have suggested that most primary EMPD express CEA, but in this study, the positive rate of CEA was only 70.5%. Therefore, GCDFP-15 and CEA still have certain limitations in diagnosing primary EMPD or distinguishing between primary and secondary EMPD. Hence, a combination of multiple immune markers is currently used to support primary EMPD diagnosis.

This study found that non-invasive primary EMPD types were predominant (76.7%), while invasive types were less common (23.3%). While this distribution aligns with findings from certain small-scale studies [8], larger-scale investigations propose a different distribution pattern for primary EMPD types with underlying malignancies (invasive type). In examining adnexal characteristics and their clinical-pathological implications, our study found adnexal involvement prevalent in 83% of cases, similar to findings from prior research advocating that involvement of adnexa is a common feature in primary EMPD [8,17], emphasizing the importance of recognizing adnexal involvement in EMPD diagnosis [18]. Furthermore, we discovered a significant link between lesion locations and adnexal involvement (p < 0.05), suggesting the importance of considering lesion sites in EMPD diagnosis and treatment. However, no significant relationships were found between adnexal involvement and demographic, clinical, or other pathological variables, indicating a need for more detailed investigations.

In exploring the prognostic implications of adnexal depth in EMPD, we performed a rank sum test of Mann-Whitney U-tests on adnexal depth and primary EMPD subtypes. The analysis revealed a significant disparity, with invasive EMPD exhibiting a deeper adnexal invasion (Mean Rank: 27.94) compared to its non-invasive counterpart (Mean Rank: 15.35, p = .001). Also, a moderate positive correlation was observed (Spearman's rho = 0.526, p = .001), indicating the likelihood of the EMPD being an invasive subtype, as adnexal depth increases, so does the prospect of EMPD being invasive. The prognosis for individuals with non-invasive primary EMPD is generally favorable as the tumor cells undergo a prolonged phase of radial growth, and most cases are managed during the carcinoma in situ stage [7,19]. In this study, although the adnexal involvement was not associated with EMPD subtypes, suggesting that adnexal involvement may not directly impact prognosis, the association between the adnexal depth and primary EMPD subtype suggests that adnexal depth can be an effective prognostic indicator.

For clinicopathological concordance assessment, dermatopathologists, blinded of clinical information, when asked to diagnose the cases identified primary EMPD as the predominant diagnosis, representing 81.4% (35 out of 43 cases). Paget-like Bowen's disease and malignant melanoma were less common, constituting 11.6% (five cases) and 7.0% (three cases), respectively. A significant difference was observed in clinicopathological concordance rates, with adnexal involvement showing a 58.3% rate compared to 14.3% in cases without adnexal involvement (p < 0.05). This finding suggests that adnexal involvement may have a notable impact on the accuracy of clinical diagnoses when correlated with pathological diagnosis, aligning with previous studies [20]. The results highlight the importance of considering adnexal involvement in the diagnostic process of diseases with clinicopathological correlations, potentially improving diagnostic precision and patient management strategies.

In our cohort, the three-year recurrence rate of EMPD cases was 44.2% (19 cases), falling within the previously reported range for postoperative recurrence rates, which vary from 31% to 61% [21,22], and the mean recurrence time was 16.6 months. This aligns with other studies suggesting long-term recurrence is a characteristic feature in primary EMPD regardless of treatment method [15]. Despite surgical excision being the standard therapeutic approach, the high variability in recurrence rates underscores the complexity of managing this condition and highlights the need for a deeper understanding of the factors driving local recurrence. Interestingly, our analysis revealed that patients with smaller lesions (≤10 cm²) had a longer mean recurrence-free survival of 31.2 months compared to 23.3 months for those with larger lesions (>10 cm²), suggesting lesion size as a potential prognostic indicator in EMPD. This finding contrasts existing literature with no definitive association between lesion size and recurrence rates [21], indicating the need for further investigation into the complexities of EMPD progression and management. Further, patients with invasive EMPD had a notably shorter mean recurrence-free interval of 17.6 months compared to those with non-invasive forms of the disease, a finding that is consistent with the observed significant difference in recurrence rates between intraepithelial and invasive EMPDV in other studies [22]. This alignment of results underscores the critical prognostic impact of Pagetoid infiltration level on recurrence, emphasizing the need for aggressive monitoring and management strategies in invasive cases.

Our study's multivariate analysis underscores the significance of the invasion level in primary EMPD as the sole independent predictor of recurrence, with invasive EMPD patients facing a recurrence risk approximately 7.6 times higher than their non-invasive counterparts, in concordance with a similar scale study. Other covariates were not significant predictors of recurrence outcome. Recent literature on EMPD treatment modalities and their impact on recurrence presents varied conclusions, with several studies finding treatment approaches not significantly affecting recurrence rates [23]. This aspect, however, was not explored in our study, highlighting a potential area for future research to understand how different treatments influence EMPD recurrence outcomes. Our study, as a single-centered, relatively low sample size, retrospective analysis, potentially limits the broader applicability of our findings. The unique treatment protocols and follow-up strategies of our center may not fully represent the generalizability of approaches in managing EMPD. The variability in follow-up duration among our study subjects might have also influenced the observed outcomes, particularly regarding disease recurrence and survival rates. Addressing these limitations in future studies could enhance the robustness and applicability of the findings.

## Conclusions

Clinical misdiagnosis is common, often due to asymptomatic or mimicking presentations. Pathologically, adnexal involvement is prevalent and significantly correlated with invasive EMPD subtypes. Deeper adnexal invasion is associated with invasive EMPD, emphasizing its importance in diagnosis and prognosis. Immunohistochemical markers are valuable for diagnosing EMPD, though distinguishing between primary and secondary types remains challenging. High recurrence rates, especially in invasive cases, highlight the need for diligent monitoring and management. Overall, adnexal proliferation in primary EMPD is vital for clinicopathological correlations and improving diagnostic accuracy, underscoring its utility in both diagnosis and prognosis.
